# Physical activity promotion among pregnancy – the role of physician from the women’s perspective

**DOI:** 10.3389/fpubh.2024.1335983

**Published:** 2024-02-29

**Authors:** Ida Laudańska-Krzemińska, Jana Krzysztoszek

**Affiliations:** ^1^Department of Physical Activity and Health Promotion Science, Poznan University of Physical Education, Poznan, Poland; ^2^Department of Didactics of Physical Activity, Poznan University of Physical Education, Poznan, Poland

**Keywords:** physical activity, exercise, pregnancy, advice, counselling, practitioner, physician

## Abstract

**Objective:**

The clear benefits of planned and supervised physical activity (PA) during pregnancy make it imperative that women are encouraged and educated about this activity. This study aimed to investigate how effectively physician promote physical activity and exercise among pregnant women. It also examines pro-health changes in selected health behaviours during pregnancy.

**Methods:**

This cross-sectional study recruited a total of 353 pregnant women in Wielkopolskie Voivodship in Poland. An anonymous survey (on-line or in-paper) was used to assess physical activity before and during pregnancy (with Pregnancy Physical Activity Questionnaire), physical activity self-efficacy, well-being (WHO-5 Well-Being Index), and guidance received from physicians on physical activity during pregnancy.

**Results:**

Only 41% of women surveyed followed WHO recommendations for PA before pregnancy, and they were much more likely to discuss safety and the need to change the intensity or type of PA with their doctor or gynaecologist. Only 23% of women were asked about their PA before pregnancy and less than 40% were encouraged to be active during pregnancy. We observed a higher probability of poor well-being among pregnant women who were inactive before pregnancy (OR = 1.873, 95%CI 1.026 to 3.421, *p* = 0.041).

**Conclusion:**

Health professionals infrequently educate and motivate women to be physically active during pregnancy. Physician advice, as it is currently perceived by women, seems to be insufficient to help pregnant women meet the recommendations for PA during pregnancy.

## 1 Introduction

Pregnancy is a unique and thought-provoking time in a woman’s life. It is also a “teachable moment” for positive changes in health behaviour (1) that will benefit the health of the developing child in her womb. There is no longer any doubt about the need for women to engage in physical activity (PA) both before and during a pregnancy, as long as it is a normal pregnancy.

For the past two decades, various national and international opinion leaders have been issuing increasingly detailed recommendations on PA during pregnancy, emphasizing its beneficial effects on the health of both the woman and the new-born. One of the first more detailed guidelines was the recommendation of the American College of Obstetricians and Gynaecologists published in 1985 (2), revised in 1994 (3) and updated in 2002 (4). Their update clarified that a pregnant woman’s health training should be repeated at least 3 times a week or more often (5). These recommendations were detailed by the Centers for Disease Control and Prevention (CDC), which recommends that pregnant women should spend at least 150 min per week on moderate aerobic exercises, for example, fast marching, gardening, swimming, and other exercises that involve large muscles groups and increase the heart rate (6). Many other organizations around the world, such as the U.S. Department of Health and Human Services (7), Sports Medicine Australia (8), The Royal Australian and New Zealand College of Obstetricians and Gynaecologists (9), and the UK Department of Health and Social Care (10), have similar views on PA during pregnancy. Also, after reviewing the latest scientific evidence regarding the relationship between PA and health, experts from the Guideline Development Group published new World Health Organization (WHO) recommendations on PA in 2020, including for pregnant women. The WHO recommends that all pregnant and postpartum women without medical contraindications engage with regular PA and perform at least 150 min of PA of moderate intensity per week, as well as aerobic, muscle-strengthening, and gentle stretching exercises (11). In this context, the current position of the Polish Society of Gynaecologists and Obstetricians (PTGiP) until 2023 was surprising to say the least. The PTGiP recommended lowering PA levels in women with uncomplicated pregnancies and described undertaking or increasing it as contraindicated (12). It is immensely gratifying to see the new recommendations published by PTGiP (13). PTGiP and PTMS (Polish Society of Sports Medicine) have created detailed guidelines for doctors, midwives, coaches, and physiotherapists regarding recommended PA for women, including those with various dysfunctions and pregnancy-related contraindications, based on the latest recommendations of international institutions and Evidence-Based Guidelines (14). Recommended activities include endurance, resistance, stretching exercises, neuromotor exercise training, and pelvic floor training for previously inactive and active women, broken down by age.

Physical activity in pregnant women stimulates the whole body and has multiple both short- and long-term benefits for maternal and fetal health (15–19). Moderate-intensity physical activity during pregnancy can decrease the likelihood of excessive weight gain, gestational diabetes, and postpartum depression symptoms (20). Physical activity is also one of the most important protective behaviours against poor well-being, which affects at least 25% of pregnant women, especially those with less education and social support (21, 22). In addition to alleviating pregnancy-related symptoms, physical activity during pregnancy can also reduce low back pain, prevent urinary incontinence and fetal macrosomia, increase joint mobility, improve circulatory function by strengthening the heart and blood vessels, and reduce the risk of hypertension and pre-eclampsia (23, 24). Two meta-analyses found no or a small effect of leisure-time physical activity on mean birth weight, and no differences in low birth weight or mean birth weight between the exercise and control groups (20, 23). Regular physical activity during pregnancy promotes healthy weight gain, aids in postpartum weight loss, and facilitates a rapid return to a state of general fitness (20, 24). PA is proposed as a preventative or therapeutic measure to reduce pregnancy complications and optimize maternal–fetal health (25).

Despite the proven benefits of physical activity for the health of pregnant women, many are not properly advised and concerns about potential risks contribute to the abandonment or refusal to exercise during this period. According to a national study by Banys et al. (26), up to 46% of women surveyed reduced their PA after becoming pregnant. It should also be added that these women were moderately physically active before becoming pregnant. Meanwhile, Wojtyła et al. (27) report that in a study of 3,451 Polish women, more than 60% of them reported limiting PA during pregnancy, most often due to concerns about the proper development of the foetus.

According to Atkinson et al. (28), there are many challenges to practicing physical activity during pregnancy. This include: women’s lack of knowledge about existing recommendations, lack of knowledge about how to engage in physical activity, lack of social support, and unavailability of physical activity opportunities. After the assessment of potential contraindications for exercising, healthcare providers should offer counselling on an active lifestyle and refer pregnant women to a qualified exercise professional (i.e., exercise physiologist or prenatal exercise specialist) with a background and experience in pregnancy and/or postpartum physical activity and/or exercise (29). The evident benefits of planned and supervised PA during pregnancy make it imperative that women are encouraged and educated about such activity. This is especially true in view of the apparent hypokinesia of modern man in the 21st century (sedentary lifestyle, passive leisure, and passive recreation). An expectant mother should be aware that her health-promoting behaviour affects both her health and that of her baby. A positive relationship was observed between mothers’ knowledge of PA during pregnancy and their daily PA (30). Hence, the role of health professionals (doctors, midwives, physiotherapists) is so important, and why they should form habits in women that affect the proper course of pregnancy. This shows the high expectations placed on this group of professionals in terms of their educational role, detailed knowledge, and willingness to cooperate in PA with both perinatal women and other maternity care providers.

The study assesses how well physicians and gynaecologists promote physical activity among pregnant women. It also examines pro-health changes in physical activity, smoking, and alcohol consumption during pregnancy. Our hypothesis suggests health professionals fail to meet expectations for promoting physical activity during pregnancy and that pregnancy is a time for pro-health changes.

## 2 Methods

### 2.1 Study design and study population

We conducted cross-sectional study among 353 pregnant women in Wielkopolskie Voivodship in Poland from 2018 to 2022. The women were over 18 years old and willing to complete an anonymous survey (on-line or in-paper). Women were recruited using the snowball method among pregnant women in maternity clinics, at birthing schools, on classes for pregnant women, at hospitals. In addition, a web-based online survey for pregnant women was used, which was promoted in forums for expectant mothers.

### 2.2 Measurements

#### 2.2.1 Physical activity and self-efficacy in physical exercise

Anonymous questionnaires were used in the research. Physical activity (PA) level before pregnancy was assessed using two questions based on WHO recommendations regarding accumulation of at least 150 min of moderate physical activity or 75 min of vigorous physical activity, or a mix of the two, per week. We asked about the number of days with at least 30 min per day of moderate or 20 min per day of vigorous physical activity in the average week before pregnancy. Self-perceived changing in PA during pregnancy were also assessed by asking the question: “Did your physical activity change after you became pregnant?,” where the respondent had five possible answers on a Likert scale from “at a much lower” to “a much higher.” PA during pregnancy was assessed using the Polish version of the Pregnancy Physical Activity Questionnaire (PPAQ-PL) (31). It consists of 33 items grouped into the following activity categories: household/caregiving (15 items), occupational (5 items), sports/exercises (7–9 items), transportation (3 items), and inactivity (3 items). The levels of PA were measured as energy expenditure (MET minutes/week). According to the authors’ guidelines the following activity intensity ranges were used: sedentary <1.5 METs; light 1.5 – < 3.0 METs; moderate ≥3.0 – ≤ 6.0 METs; and vigorous >6.0 METs (32).

A questionnaire developed by Schwarzer and Renner (33) was used to assess health–specific self-efficacy The Physical Exercise Self-Efficacy Scale includes five statements referring to the potential obstacles to carrying out exercises by respondents. The four responses were proposed for all statements from “very uncertain” to “very certain”.

#### 2.2.2 Well-being and health behaviours

The World Health Organization- Five Well-Being Index (WHO-5) was used to assess current mental health and well-being (34). According to the authors’ recommendation, a score of less than 13 indicates poor well-being and is an indication for testing for depression according to ICD-10. Self-perceived changes in smoking and alcohol consumption during pregnancy were also assessed by asking the question: “Your contact with cigarettes/alcohol before and after pregnancy,” where the respondent had three possible answers for smoking before pregnancy and the same during pregnancy: “I did not smoke,” “I smoked occasionally,” I smoked regularly” and four possible answers for drinking alcohol before pregnancy and the same during pregnancy: “I did not drink,” “I drank occasionally,” “I drank little regularly,” “I drank a lot regularly”.

#### 2.2.3 Physician advice on physical activity

To assess doctors’ activity in promoting physical activity among pregnant women, participants were asked whether their doctors, during consultations: (1) asked you about physical activity before pregnancy? (2) encouraged you to do the physical activity you needed during pregnancy? (3) stated that you had no health contraindications to undertake PA? (4) You were the first who ask for advice on PA during pregnancy? (5) Has your doctor recommended a modification of your current PA? If so, what is it: duration, frequency, intensity, or type of physical activity? The questionnaire was developed by an interdisciplinary team of experts including a gynaecologist, a physiotherapist, a medical trainer, and an exercise specialist. Then, it was tested for comprehension and clarity of wording by 6 pregnant women. The consistency of responses from 30 pregnant women was also assessed by analysing the test and retest results. For the 5 analysed questions the Cohen’s kappa scores ranged from 0.80 to 1.00, indicating almost perfect agreement and good reliability of the tool (35).

#### 2.2.4 Pregnant’ characteristics

Sociodemographic characteristics included maternal age, gestational age, height, pre-pregnancy and current weight, body height, educational level, place of residence, number of pregnancies.

### 2.3 Statistical analysis

The calculations were performed using STATISTICA 13.3 (StatSoft, Inc.). The characteristics were shown as mean ± standard deviation (SD), medians, and mean rank or as proportions if variables were categorical. To evaluate differences in self-efficacy, well-being, BMI and physical activity during pregnancy between active and inactive before pregnancy women the nonparametric Mann–Whitney U test was used. Effect sizes r were calculated from the test statistic z (z/sqrt (n)). Effect sizes were interpreted as small when r ≥ 0.1, medium when r ≥ 0.3, and large when r ≥ 0.5 (36). Odds ratios with 95% CI were calculated for poor well-being by physical activity before pregnancy status. To evaluate differences in the frequency of receiving information from physicians between active and inactive before pregnancy women the chi square test was used and Fi effect size was calculated, with following interpretation: as small when r ≥ 0.1, medium when r ≥ 0.3, and large when r ≥ 0.5 (36). Odds ratios with 95% CI were calculated for being ask about PA and encouraging for PA by physical activity before pregnancy status. In all tests, a *p*-value of less than 0.05 was statistically significant.

## 3 Results

We analysed the results of 353 women with a mean age of 29.3 years (SD = 4.3). There were 6% in the first, 29% in the second and 65% in the third trimester of the pregnancy. In terms of social status most of the women surveyed had a high school education (69%), followed by secondary education (24%) and vocational education (7%), and lived in a big city (52%), followed by a small city (27%) and countryside (21%). For 55% of respondents this was their first pregnancy, for 28% it was their second pregnancy and for 17% it was more than second pregnancy (see Table 1).

**Table 1:** General studied sample characteristics (*N* = 353).

Variables	Mean	SD	Median
Age (years)	29.3	4.3	29
BMI before pregnancy (kg/m^2^)	22.5	3.7	21.6
Self-efficacy in PA^a^ (pts)	13.2	4.2	13
Well-being WHO-5^b^ (pts)	15.2	5.0	15
Total PA during pregnancy^c^ (METs)	167.81	87.25	147.61
Physical activity during pregnancy by intensity^c^ (METs)			
Sedentary	25.92	21.08	18.90
Light	92.72	52.50	80.68
Moderate	48.03	47.44	32.80
Vigorous	1.14	2.93	0.00
Physical activity during pregnancy by type^c^ (METs)			
Household	78.27	64.98	344.27
Occupational	23.12	47.62	0.00
Sport	13.20	13.71	9.52
Transportation	17,0.19	18.44	12.60
Inactivity	36.032	25.26	31.50

a The Physical Exercise Self-Efficacy Scale, score ranging from 5 to 20.

b Five Well-Being Index (WHO-5), score ranging from 0 to 25.

c Pregnancy Physical Activity Questionnaire (PPAQ-PL) in MET minutes/week.

According to the level of PA before pregnancy, followed by the WHO recommendation, we divided the studied women into two groups: active (reported at least 5 days with 30 min of moderate-intensity aerobic physical activity or at least 3–4 days with 20 min of vigorous-intensity aerobic physical activity or an equivalent combination of moderate- and vigorous-intensity activity throughout the week). In terms of the studied group declaration, 41% of the women had sufficient physical activity. The differences in the analyzed parameters between active and inactive women before pregnancy are shown in Table 2.

**Table 2:** Biopsychosocial characteristics and PA during pregnancy for physical active and inactive women before pregnancy.

Variable	Active before pregnancy (*n* = 146)			Inactive before pregnancy (*n* = 207)			*p*	*r*
	x (SD)	Median	Mean rank	x (SD)	Median	Mean rank		
BMI before pregnancy	22.4 (3.9)	21.3	181.79	22,5 (3,6)	21,9	169.04	0.247	−0.062
Self-efficacy in PA^a^	14.8 (3.6)	15	174.86	12.0 (4.1)	12	119.16	**<0.0001**	0.300
Well-being WHO-5^b^	16.1 (4.44)	16.5	121.21	14.44 (5.27)	15	101.25	**0.021**	0.123
Total PA during pregnancy^c^ (METs)	180.46 (92.82)	158.63	189.52	158.98 (82.36)	140.75	165.58	**0.029**	0.116
Physical activity during pregnancy by intensity^c^ (METs)								
Sedentary	25.95 (19.70)	18.90	179.42	25.94 (22.08)	18.90	172.72	0.542	0.033
Light	96.90 (56.19)	80.46	181.75	89.76 (49.79)	80.68	171.08	0.331	0.052
Moderate	55.70 (50.93)	39.29	193.20	42.69 (44,26)	27.56	162.98	**0.006**	0.146
Vigorous	1.91 (4.19)	0.00	186.12	0,60 (1.28)	0.00	167.99	0.099	0.088
Physical activity during pregnancy by type^c^ (METs)								
Household	78.12 (65.96)	55.65	172.98	78.39 (64.60)	61.25	177.29	0.695	−0.020
Occupational	25.81 (53.33)	0.00	175.5	21.33 (43.27)	0.00	175.5	0.999	0.000
Sport	18.24 (15.36)	13.45	216.79	9,63 (11.17)	6.19	146.34	**<0.0001**	0.342
Transportation	21.09 (24.47)	14.00	191.68	14.40 (11.92)	10.71	164.05	**0.012**	0.134
Inactivity	37.21 (23.82)	35.70	183.77	35.23 (26.32)	30.45	169.65	0.199	0.068

a The Physical Exercise Self-Efficacy Scale, score ranging from 5 to 20.

b Five Well-Being Index (WHO-5), score ranging from 0 to 25.

c Pregnancy Physical Activity Questionnaire (PPAQ-PL) in MET minutes/weekBold values are *p*-value < 0.05.

There was no difference in BMI between active and inactive women before pregnancy. We found that active woman had significantly higher levels of PA self-efficacy and well-being. According to the recommendation of the Psychiatric Research Unit WHO Collaborating Centre in Mental Health (37), 30.0% of the women surveyed present poor well-being, which is an indication for testing for depression according to ICD-10. It also implies a lower risk of depression among women who were physically active before pregnancy. We observed a higher probability of poor well-being among women who were inactive before pregnancy (OR = 1.873, 95%CI 1.026 to 3.421, *p* = 0.041). Higher self-efficacy in PA allows people to set, maintain, and achieve daily routines. Physically active women before pregnancy have significantly higher levels of total PA measurement during pregnancy (*p* = 0.029; *r* = 0.116) and those related to sports activities and transport during pregnancy (*p* < 0.0001, *r* = 0.342 and *p* = 0.012, *r* = 0.134 respectively). This is also related to the higher levels of moderate PA among active women surveyed before pregnancy (*p* = 0.006, *r* = 0.146).

We then analysed if and how the level of physical activity before pregnancy was related to the willingness to discuss safety and necessary modification of intensity or form of physical activity with a physician or gynaecologist. The results are presented in Table 3.

**Table 3:** Frequency of consultations and interviews on PA with doctors and gynaecologists – differentiation between active and inactive women before pregnancy (results of chi-square test).

Doctors’ questions/ advice on^a^:	All (*n* = 353) yes (%)	Active before pregnancy (*n* = 146) yes (%)	Inactive before pregnancy (*n* = 207) yes (%)	*p*	Fi
PA before pregnancy	23.6	29.5	19.4	**0.029**	0.116
Encouraging to PA	37.5	43.8	33.0	**0.039**	0.110
Contraindications to PA	54.3	60.3	50.0	0.057	0.102
Women first ask for a consultation on PA	41.2	56.2	32.2	**<0.0001**	0.239
The doctor recommends modification of the PA	30.7	43.2	21.8	**<0.0001**	0.228
Modification of time*	44.4	46.6	41.5	0.616	0.050
Modification of frequency	38.1	37.5	39.0	0.879	0.016
Modification of intensity	77.1	81.9	70.5	0.166	0.135
Modification of form	77.2	78.3	75.6	0.749	0.032

*Percentage of those who answered “yes” to the previous question.

a Physician advice on physical activity (dichotomous answer: yes/no).Bold values are *p*-value < 0.05.

More than 50% of pregnant women admit that their gynecologist has told them there is no contradiction to PA. At the same time, only 23% of women were asked about PA before pregnancy and less than 40% were encouraged to be active during pregnancy. Women who were active before pregnancy were asked about PA and encouraged to be active more often (*p* = 0.029; and *p* = 0.039 respectively). We observed a higher likelihood of being asked about PA before pregnancy (OR = 1.733, 95%CI 1.055 to 2.845, *p* = 0.030) and being encouraged to be active during pregnancy (OR = 1.584, 95%CI 1.023 to 2.453, *p* = 0.039) for women who were active before pregnancy. At least 40% respondents asked for advice on PA first. Physically active women were more likely to do so (*p* < 0.0001). Around 30% of the women surveyed had received recommendations for PA modifications from their doctors, more often active ones (*p* < 0.0001). Doctors’ advice was most often related to modifying the intensity and type of PA, less often about frequency and time.

Physical activity before pregnancy did not differentiate the behaviours related to smoking or alcohol consumption (Table 4). Before pregnancy 11% of the women analysed smoked regularly, 17% occasionally, and after getting pregnant respectively: 1.2% and 3.2%. Similarly, 10% of the women analysed drank alcohol regularly before pregnancy and almost 70% occasionally and after getting pregnant: 0,8% and 4,5%, respectively. Regarding declarations of changes in physical activity during pregnancy 61% of the women surveyed declare that their PA had decreased a lot or a little, and only 13% declared that their PA had increased a little or a lot. The rest (26%) did not change their PA level (Table 5).

**Table 4:** Risky behaviour among active and inactive before pregnancy respondents.

Variable	All (*n* = 353) yes (%)	Active before pregnancy (*n* = 146) yes (%)	Inactive before pregnancy (*n* = 207) yes (%)	*p*	Fi
Smoking before pregnancy (occasionally or regular)^a^	28.2	25.0	30.1	0.381	0.056
Smoking in pregnancy (occasionally or regular)^a^	4.4	3.8	4.9	0.693	0.025
Drinking alcohol before pregnancy^b^	79.1	84.6	74.8	0.062	0.119
Drinking alcohol during pregnancy^b^	5.2	5.8	4.9	0.761	0.019

a Smoking regularly or occasionally (yes), do not smoke (no).

b Drinking alcohol occasionally, a little regularly or a lot regularly (yes), do not drink (no).

**Table 5:** Respondents’ declaration of changes in risky behaviours and PA during pregnancy.

Variable	Regularly (%)	Occasionally (%)	No (%)	
Smoking before pregnancy	11.3	16.9	71.8	
Smoking during pregnancy	1.2	3.2	95.6	

## 4 Discussion

Our study identified a weak link in the path toward meeting PA recommendations for pregnant women. Healthcare professionals do not often educate and motivate women to be physically active during pregnancy. This can be easily and cost-effectively changed with health benefits for mothers and children and financial benefits for health care payers.

In our study, far less than half of the surveyed women had received advice about being active during pregnancy. Santo et al. (38) also found that almost a third did not receive advice on physical activity during prenatal care. Obese women were no more likely to receive advice than their normal weight counterparts, indicating the need for targeted physical activity counselling in this population. Similarly, Whitaker et al. (39) found that only around 60% of women surveyed reported receiving advice on physical activity from their provider, which, although consistent with guidelines, was perceived by patients to be limited in scope. In the study by Beckham et al. (40), the authors also found that women did not receive sufficient and clear information about how and why to exercise during pregnancy. Counselling rates during preventive care visits for women of childbearing age vary by overweight/obesity and pregnancy status, as well as by provider specialty (41). Although healthcare practitioners may be in unique position to provide exercise advice to pregnant women, they may not have the necessary knowledge, training, or support to provide specific exercise advice (42).

As the results of our study showed, less than half of the women surveyed had a healthy lifestyle before becoming pregnant, and respondents with more education were more aware of the importance of this factor, including regular exercise. For most pregnant women, engaging in moderate-intensity PA has few risks but many health benefits, including a reduced risk of gestational diabetes and postpartum depression (14). The results of a survey of a population of Polish women by Szatko et al. (43) show that a total of 92.5% of women were aware of the beneficial effects of PA on the course of an uncomplicated pregnancy. Pregnant women were aware that PA reduces the risk of gestational diabetes mellitus and pre-eclampsia, and that moderate exercise reduce the likelihood of operative labour. Higher education was associated with greater awareness (*p* = 0.001). The most common sources of information on PA during pregnancy were the internet (50.0%) and books (38.3%). Doctors and midwives instructed the respondents only in 22.4 and 18.9% of cases, respectively. In our survey, the percentages were slightly higher (encouraging PA 37.5%, recommending for PA 30.7%), but these results indicate that health professionals are not the dominant source of information about PA in pregnancy. A study by Torbè et al. (44) found that only 2% of the 100 pregnant women surveyed identified their pregnancy doctor as a source of information about exercise during pregnancy. Doctors need to strengthen their role as providers of reliable and high quality of information, which would lead to the prevention of many pregnancy complications. The second essential condition for continued physical activity at any stage of life, including pregnancy, is high motivation. This is a condition that should also be address by health professionals, countering family stereotypes. In the analysis by Findley et al. (45), women with a history of pregnancy reported that family members and partners advised them to stop exercising during pregnancy and offered them advice, leading them to a perceived a lack of ownership of their bodies. Studies have shown that well designed lifestyle counselling, provided as part of routine care, leads to improvements in the PA patterns in pregnant women (46–48), and should therefore be a mandatory part of all antenatal and obstetric consultations.

It is noteworthy that in our study such discussions and advice were most often recorded with women who were active before pregnancy, and it was them who initiated such consultations on physical activity, indicating the real passivity of doctors in this regard. Women’s attitude can be explained by the results of the study by Moreno et al. (49), which showed that those who regularly engage in some form of physical activity or sport are more interested in exercise and have more positive view of their physical fitness than those who do not engage in sport at all.

It is worth considering the involvement of other professionals who may be caring for the pregnant woman in promoting healthy behaviours. Specialists who promote healthy physical activity habits include physiotherapists. They are the ones that pregnant women turn to for help with back and/or musculoskeletal pain caused by changes in the body during pregnancy. As Sapuła et al. (50) state, in addition appropriate physiotherapeutic interventions, the formation of health-promoting attitudes in patients is an important task for physiotherapists. In particular, attention and efforts should be focused on inactive women in order to mobilise them, as the current state of affairs is contrary to the recommendations.

Influencing pregnant women in this area is also extremely important for another reason. We have shown that active women have a significantly higher levels of PA self-efficacy and well-being than inactive women before pregnancy. Moreno-Murcia et al. (51) came to similar conclusions, stating that engaging in physical activity and sport in general includes several activities and elements that are present in the well-being and satisfaction of physically active women. These elements evoke positive emotions that enable women to achieve the goals they have set for themselves. As a result, they remain active during pregnancy and are less affected by poor well-being, which can contribute to postpartum depression. The systematic review by Liu et al. suggests that group-based combined exercise and yoga or PA are associated with significant benefits for the quality of life of pregnant women (52). Systematic reviews and meta-analyses by Gong et al. (53) and Lin et al. (54) found that prenatal yoga can effectively reduce depressive symptoms in pregnant women. The positive association between exercise and the prevention of maternal prenatal depression has been demonstrated in some studies with supervised exercise programs and high participant adherence (55, 56). Another systematic review and meta-analysis by Sánchez-Polán et al. (57) also concluded that supervised exercise during pregnancy is and effective tool for preventing and reducing prenatal depression. Of particular note is the study conducted by Perales et al. (58). The study involved 167 expectant mothers and found that while the level of depression was similar in both groups at the beginning of pregnancy, there was a significant difference at the end of pregnancy. The intervention group, which underwent supervised exercise programs, experienced a reduction in depression levels compared with the control group. This result confirms the positive effect of a supervised exercise program during pregnancy on the emotional state of pregnant women. These findings suggest that there is a positive association between an active pregnancy and a more balanced and appropriate emotional state. The effects of exercise during pregnancy may be a beneficial approach to alleviating prenatal depression and promoting the overall well-being of both mothers and their unborn children. Before starting any exercise regimen, pregnant women should see a health professional for personalized advice and professional information support (14). Given the proven protective properties of physical activity in this area, it is worth increasing the promotion of physical activity for this reason alone.

We observed a decrease in anti-health behaviours among pregnant women. Regular smoking decreased by 9.8 percentage points and occasional smoking decreased by 13.8 percentage points. Regular alcohol consumption decreased by 9.2 percentage points, and occasional consumption decreased by 65.5 percentage points. Similar changes have been observed by other researchers as well. In a study by Scheffers-van Schayck et al. (59), about 40% of pregnant smokers quit smoking during pregnancy. Similarly, Jawad et al. (60) found that, overall, women reported improved health behaviours during pregnancy, such as reducing or quitting smoking, drinking alcohol, and taking dietary supplements. These changes indirectly demonstrate the effectiveness of clinicians in reducing these behaviours. In 1999, Jones-Webb et al. (61) conducted a study of the direct effect of medical advice on tobacco and alcohol use during pregnancy. The study found that pregnant women who received advice from their doctors about the risks of using these substances were more likely to abstain. This suggests that medical advice can be a powerful motivator for healthier behaviour during pregnancy (61). Looking more broadly at prenatal care, Evans and Sheu’s validation of the adherence pathway model found that effective patient-physician communication was a key factor in promoting adherence among pregnant women (62).

Given the past effectiveness of health professionals in minimizing harmful behaviours in pregnant women, it would be reasonable to expect similar positive outcomes from routine interactions about physical activity. Women in particular often report a lack of such advice (in terms of PA) (63), which is largely limited to walking. Our research also points to deficiencies in this area. Many women in the Ferrari et al. study (63) reported that they followed the advice, and if they did not, it was because the women disagree with the advice or simply did not want to follow it. This may be the result of not having enough well-explained information that is clear and convincing. Existing research shows that UK medical students underestimate the risk of physical inactivity and did not know the physical activity guidelines (64). Other research shows that although medical students are generally active and have a good understanding of the links between PA and health, they lack skills in PA counselling. Improved education of this group is required (65). Therefore, one of the reasons for the low involvement of antenatal physicians in the promotion of physical activity may be their insufficient education in this area during their studies and gynaecological specialization. Now that new, modern recommendations in this area have also been published in Poland (13), this important aspect of education and health promotion for pregnant women should be addressed more intensively at medical universities (66). Medical advice is an essential component of prenatal care, helping pregnant women to make informed decisions to protect both their health and the health of their unborn child. It serves as a powerful tool in reducing tobacco and alcohol use during pregnancy, ultimately contributing to better pregnancy outcomes. It would be worthwhile for physicians to make PA advice more transparent and more personalized, to provide it repeatedly during pregnancy in an understandable and persuasive way, and to take the time to do so. One of the good practices in this area is the intervention strategy to promote prenatal physical activity proposed in the Buffalo City Municipality, Eastern Cape Province, South Africa, where all stakeholders were involved in the creating and development process (67). The medical community is strongly supported by numerous public campaigns and media activities in this area. Promoting physical activity with such tools could also make a significant contribution.

We believe that a strength of our study is the use of validated tool to assess physical activity of pregnant women and the attempt to assess the quality of doctor-patient communication regarding physical activity recommendations. We also recognise the limitations associated with the recruitment to the study. Although it was carried out in a variety of locations, it can be assumed that women who were interested in an active lifestyle during pregnancy were more likely to participate, hence some results may be overestimated. We also believe that a more complete picture would be obtained by surveying doctors themselves in a similar area.

## 5 Conclusion

In conclusion, one of the weak links in Poland in meeting PA recommendations for pregnant women is the antenatal care provider. Doctors rarely educate and motivate women to be physically active during pregnancy. Doctors’ advice, as currently perceived by women, does not appear to be sufficient to help pregnant women meet the recommendations for PA in pregnancy. More research is needed to better understand why so few women are physically active during pregnancy. The majority of respondents reported positive changes during pregnancy in terms of risky behaviours (alcohol consumption and smoking). This was not the case for physical activity, which decreased, indicating an area where education, including counselling provided by doctors, is necessary and essential. Although women’s reports are crucial to understand PA behaviour during pregnancy, self-reports alone are not sufficient. Future research would benefit from including providers’ perspectives and contrasting these with women’s reports. Gathering information from providers should reveal barriers to better understanding of advice and, ideally, guide when and how to intervene to promote optimal PA among pregnant women.

## Data availability statement

The raw data supporting the conclusions of this article will be made available by the authors, without undue reservation.

## Ethics statement

The studies involving humans were approved by Bioethics Committee at Poznań University of Medical Science. The studies were conducted in accordance with the local legislation and institutional requirements. The participants provided their written informed consent to participate in this study.

## Author contributions

IL-K: Conceptualization, Formal analysis, Investigation, Methodology, Writing – original draft, Writing – review & editing. JK: Conceptualization, Writing – original draft, Writing – review & editing.
